# Diagnosis of Pancreatic Ductal Adenocarcinoma Using Deep Learning

**DOI:** 10.3390/s24217005

**Published:** 2024-10-31

**Authors:** Fulya Kavak, Sebnem Bora, Aylin Kantarci, Aybars Uğur, Sumru Cagaptay, Deniz Gokcay, Anıl Aysal, Burcin Pehlivanoglu, Ozgul Sagol

**Affiliations:** 1 Department of Computer Engineering, Ege University, 35040 Izmir, Turkey; fulya-kavak@outlook.com (F.K.); aylin.kantarci@ege.edu.tr (A.K.); aybars.ugur@ege.edu.tr (A.U.); 2 Department of Pathology, Faculty of Medicine, Dokuz Eylul University, 35220 Izmir, Turkey; sumru.cagaptay@deu.edu.tr (S.C.); deniz.kabakci@deu.edu.tr (D.G.); anil.aysal@deu.edu.tr (A.A.); burcin.pehlivanoglu@deu.edu.tr (B.P.); ozgul.sagol@deu.edu.tr (O.S.)

**Keywords:** deep learning, convolutional neural networks, classification, health services, pathology images, pancreatic ductal adenocarcinoma

## Abstract

Recent advances in artificial intelligence (AI) research, particularly in image processing technologies, have shown promising applications across various domains, including health care. There is a significant effort to use AI for the early diagnosis and detection of diseases, offering cost-effective and timely solutions to enhance patient outcomes. This study introduces a deep learning network aimed at analyzing pathology images for the accurate diagnosis of pancreatic cancer, specifically pancreatic ductal adenocarcinoma (PDAC). Utilizing a novel dataset comprised of cases diagnosed with PDAC and/or chronic pancreatitis, this study applies deep learning algorithms to assess the effectiveness and reliability of the diagnostic process. The dataset was enhanced through image duplication and the creation of a second dataset with varied dimensions, facilitating the training of advanced transfer learning models including InceptionV3, DenseNet, ResNet, VGG, EfficientNet, and a specially designed deep neural network. The study presents a convolutional neural network model, optimized for the rapid and accurate detection of pancreatic cancer, and conducts a comparative analysis with other models to select the most accurate algorithm for a decision support system. The results from Dataset 1 show that EfficientNetB0 achieved a high success rate of 92%. In Dataset 2, VGG16 was found to have high performance, with a success rate of 92%. On the other hand, ResNet50 achieved a remarkable success rate of 96% despite a moderate training time and showed high precision, recall, F1 score, and accuracy. These results provide valuable data to demonstrate and share the relevance of different deep learning models in pancreatic cancer diagnosis.

## 1. Introduction

Artificial intelligence (AI) has become a main focus of research in the last decade. Today, with the rapid growth in artificial intelligence (AI) studies, interest in application areas such as object detection and classification based on image processing has increased. Medicine is one of the application domains of AI. The first purpose of health-related AI applications is to analyze the relationships between disease prevention or treatment techniques and patient treatment outcomes. 

AI software has been developed for applications such as diagnosis processes, treatment protocols, personalized medicine, patient monitoring, and patient care. Moreover, AI algorithms can be used to analyze large amounts of complex data through electronic health records for disease prevention and diagnosis. AI applications provide an opportunity to increase efficiency, especially in health care in terms of early diagnosis and cost-effective solutions. The widespread use of AI in medicine will increase even more in the coming years.

Machine learning and artificial intelligence-based studies, especially in the field of diagnosis, focus on chronic diseases such as cancer, heart diseases, Alzheimer’s, and kidney diseases and show that artificial intelligence-based applications will be encountered frequently in the diagnosis processes of these diseases in the near future. Although the results of initial studies are promising, there still seems to be a long road ahead for the daily use of AI in health care.

Over the past decade, modern machine learning methods have been successfully applied to the analysis of histopathology images thanks to significant advances in artificial intelligence and the increased ability to digitize histopathology slides. Despite the need for large amounts of labeled data, these models have demonstrated their potential in several applications, including cancer diagnosis, prognosis, and the prediction of genomic and molecular subtypes. The development of AI in this area not only facilitates more accurate and timely assessments of disease but also makes a significant contribution to clinical practice. This trend has led to the growing adoption of AI-based solutions in the histopathology field, thereby increasing the effectiveness of medical decision support systems [1].

Pancreatic ductal adenocarcinoma (PDAC) is the fourth leading cause of cancer-related deaths in the USA [2]. Pancreatic ductal adenocarcinomas (PDACs) comprise more than 90% of pancreatic cancer cases, and they exhibit a highly aggressive behavior. Chronic pancreatitis (CP) is a major diagnostic challenge for the pathologist and may mimic and accompany PDAC; distinguishing CP from PDAC is of the utmost importance for accurate diagnosis and optimal treatment. There are limited studies on the usage of deep learning in the diagnosis of pancreatic cancer [3,4].

Recently, anatomical structures within the pancreas have been classified using a deep learning algorithm on histopathological images [3]. In another study, it has been reported that pancreatic cancer can be identified by convolutional neural networks using deep transfer learning [5]. A deep convolutional neural network (DCNN) system was developed to segment cell clusters from patients from different hospitals and identify cancer cell clusters from cytopathology images [6]. Deepthi et al. [4] performed a comparative analysis of pancreatic tumor detection using VGG16, ResNet, and DenseNet. Dinesh et al. developed a system that uses convolutional neural networks (CNNs) and YOLO-based CNN (YCNN) models to identify critical features and cancerous growths in the pancreas. The study highlights the potential of the YCNN approach for accurate cancer detection and evaluates its effectiveness compared to other methods [7]. Saillard et al. developed a multi-stage deep learning model for the rapid molecular subtyping of PDAC using histopathological data. A tile-level analysis identified PDAC microheterogeneity and identified hybrid and intermediate tumor types, which could indicate transitional stages of disease progression [8].

In this study, we aimed to investigate the potential role of AI in distinguishing CP and PDAC and to develop a convolutional neural network to diagnose PDAC on histopathological images. Our study employs a specialized dataset of PDAC and chronic pancreatitis cases, enhanced through image duplication and varied dimensions, to train advanced models such as InceptionV3, DenseNet, VGG, EfficientNet, and ResNet. 

The innovations in our study are the following:

- The development of a specialized dataset containing cases of PDAC and CP is an important and innovative aspect of this study. This dataset forms the basis of the research and provides a valuable reference for future studies.
- The use of different transfer learning models in the study helps to determine the most effective model by allowing a comparative analysis of different approaches.
- The developed convolutional neural network (CNN) model is optimized for the fast and accurate detection of pancreatic cancer and has the potential to save time and costs in clinical applications.

The aim is to assess and improve the diagnostic accuracy and reliability of a decision support system. In this context, diagnostic accuracy refers to the rate at which the system provides accurate results, whereas reliability refers to the system’s ability to provide consistent and reliable results. These two concepts are critical in decision-making processes because they enable users to make decisions with confidence. Therefore, this study aims to analyze the performance of the current system and identify potential areas for improvement. 

Section 2 of this paper describes the construction of the dataset. It presents key information on the methods used and describes the training of the models. Section 3 presents the results, followed by Section 4, which discusses the results. Finally, Section 5 provides the conclusions.

## 2. Materials and Methods

The aim of this study was to perform classification with a visual dataset for pancreatic ductal adenocarcinoma (PDAC) and chronic pancreatitis (CP) analyses with the following steps:

- Data collection: Digital slides of PDAC and CP fields were photographed at 200× magnification to obtain high-resolution images.
- Diagnostic criteria: The diagnosis of PDAC was based on major criteria (e.g., 4:1 nuclear size change, incomplete ductal structures) and minor criteria (e.g., single cell infiltration, irregular stroma, mitotic figures).
- Image preparation: Two datasets were created.
- Image processing: Captured images were de-identified for privacy and systematically numbered for easy classification.
- Image characteristics: All images at 2035 × 3841 were resized for a detailed analysis. Each image was subjected to a cropping procedure to ensure a balanced dataset to obtain two different image sets, 256 × 256 pixels and 512 × 512 pixels.

This methodology provides robust datasets suitable for training deep learning algorithms to increase PDAC diagnostic accuracy and improve clinical outcomes.

### 2.1. Dataset Preparation 

**Figure 1:**
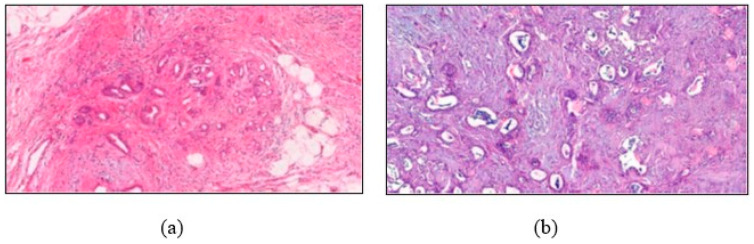
(**a**) A CP image showing the features of the dataset, and (**b**) a PDAC image showing different disease data. Both of these images are part of the datasets that were used in the study.

First, hematoxylin–eosin-stained slides from consecutive patients with PDAC and/or CP (Figure 1) diagnosed at the Department of Pathology, Dokuz Eylul University Faculty of Medicine, were re-reviewed. Representative slides were scanned with a slide scanner (3D Histech, Pannoramic 250 Flash III, Budapest, Hungary) with a 20× objective lens. On the digital slides, areas of PDAC and CP were photographed at 200× magnification to compile a comprehensive visual dataset. A nuclear size change among ductal cells of 4:1 or more, incomplete ductal structures, and the irregular distribution of ducts were the major criteria and single cell infiltration, irregular stroma, mitotic figures in epithelial cells, necrotic glandular debris, cribriform structures, large irregular eosinophilic nucleoli, and perineural invasion were the minor criteria used for the diagnosis of PDAC [9]. 

Photographs showing only malignant tumoral areas (cells or glands with these features) and photographs showing only findings of chronic pancreatitis without malignancy were prepared as two separate datasets. The images captured were de-identified at a resolution of 2035 × 3841 pixels and uniquely classified through a systematic numbering approach.

**Figure 2:**
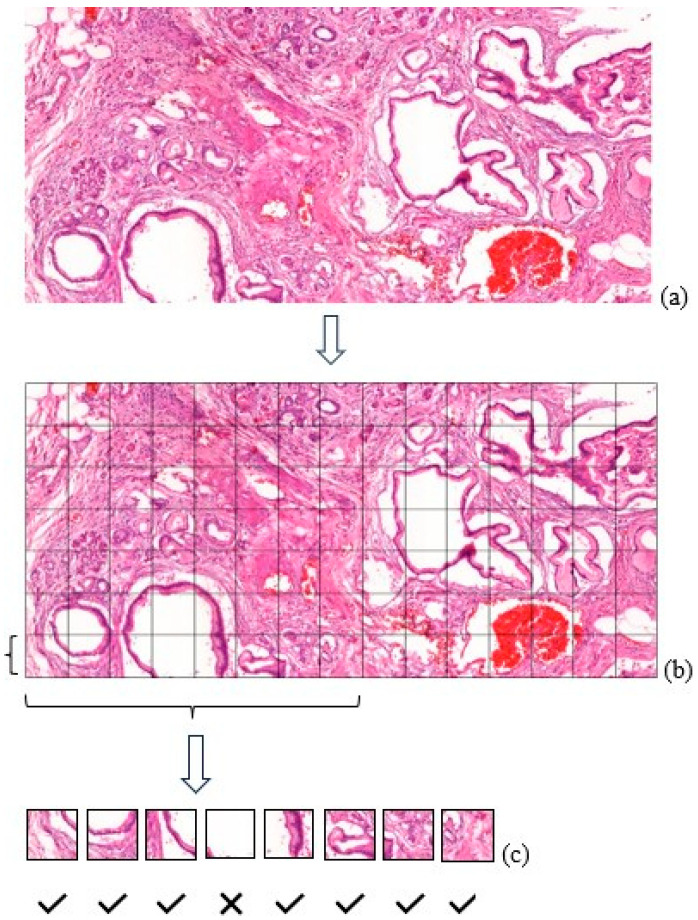
(**a**) PDAC/CP image; (**b**) the illustration demonstrates the selection of normalized tissue patches based on the CNN classification; (**c**) selected images used after the data cleanup.

A collection of 54 CP and 65 PDAC images was compiled, each with dimensions of 2035 × 3841. In order to ensure a balanced dataset, each image in the dataset was generated by a rigorous cropping procedure, resulting in two different image sets of 256 × 256 and 512 × 512 (Table 1). This process facilitated the optimal utilization of visual data for the training of CNNs, illustrated in the subsequent Figure 2.

The data was resized to two different sizes, 256 × 256 and 512 × 512. After resizing, each image was preprocessed to create a 256 × 256 Dataset 1 consisting of 12,000 images and a 512 × 512 Dataset 2 consisting of 4000 images to be used in the study.

Subsequent to cropping, the images detailed in Figure 2 were re-evaluated by a pathologist (B.P.) in order to exclude any unsuitable visuals. 

**Figure 3:**
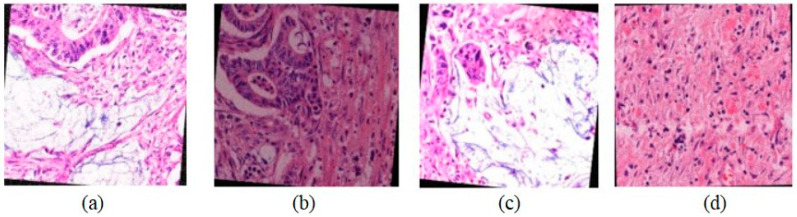
(**a**–**d**) are the images obtained as a result of image enhancement.

The images were then subjected to further augmentation—including rotation, contrast adjustments, and brightness modifications—to enrich data diversity (Figure 3).

The dataset, comprising 256 × 256 pixel images, included 6000 images each of malignant and benign areas. Similarly, in the 512 × 512 pixel dataset, both malignant and benign areas were represented by 2000 images each (Table 1). The dataset allocation consists of 80% for training, 12% for testing, and 8% for validation (Table 1). The training set underwent additional augmentation processes and background refinement to enhance data quality.

A collection of 54 chronic pancreatitis and 65 pancreatic ductal adenocarcinoma images, each 2035 × 3841 in size, were curated. To ensure a balanced dataset, two distinct sets of images were generated with dimensions of 256 256 and 512 × 512 following a meticulous cropping procedure. This process facilitated the optimal utilization of visual data for the training of CNNs, as illustrated in the subsequent figure.

The dataset, comprising 256 × 256 pixel images, includes 6000 images each of malignant and benign cells. Similarly, in the 512 × 512 pixel dataset, both malignant and benign cells are represented by 2000 images each.

The dataset allocation consists of 80% for training, 12% for testing, and 8% for validation purposes. The training set underwent additional augmentation processes and background refinement to enhance data quality (Table 2).

### 2.2. Selection of Deep Learning Techniques

To discern PDAC from CP, a range of deep learning frameworks were employed. The study focused on developing a CNN model. Following the division of the dataset into training and testing subsets, the process continued with model training and evaluation on test data.

### 2.3. Evaluation of Predictions

Following the training and testing phases, the models underwent scrutiny based on specific evaluation metrics. The analysis encompassed observing fluctuations in metrics across both balanced and imbalanced data distributions, examining the correlation between forecasted outcomes and error rates, and assessing the success metrics of the final results.

Confusion matrix, accuracy, the F1 score, loss, and precision are used as success metrics. The collection of a model allows for a more comprehensive analysis when these metrics are evaluated together.

### 2.4. Used Deep Learning Methods

In this study, different deep learning methods were used to classify PDAC and CP disease. Different types of convolutional neural networks [5] were used for feature extraction from histopathology images, species classification, and the evaluation of algorithm performance. InceptionV3 [6], DenseNet121 [4], ResNet50 [10,11], VGG19 [12], VGG16 [12,13], EfficientNetB0 [14], EfficientNetB5 [14], and the designed deep learning model were used to improve the classification performance.

### 2.5. Architecture of the Deep Learning Model

**Figure 4:**
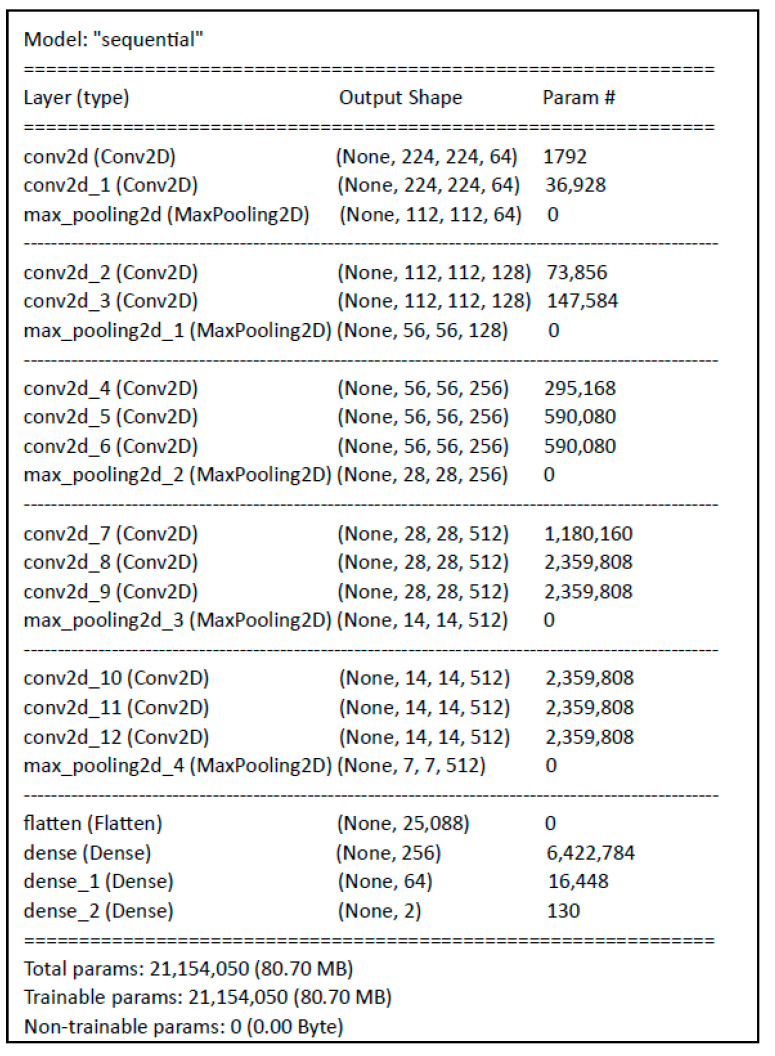
Design of the CNN architecture.

The datasets were configured in two distinct resolutions and partitioned into 80% for training purposes, with the remaining 20% allocated to testing and validation. In the constructed CNN model, the batch size was uniformly set to 64 across both datasets, and the training was conducted over 10 epochs. Following the initial preprocessing phase, a CNN was structured to facilitate the training of the image datasets, featuring an input layer dimension of 224 × 224 × 3 (Figure 4).

The constructed model adheres to the CNN framework, incorporating convolutional layers for feature extraction, max-pooling layers to reduce spatial dimensions, and dense layers to execute classification tasks. The model culminates in an output layer with two nodes, signifying a binary classification objective.

The intricate design is indicative of a model with substantial depth and complexity. In this model, convolutional layers were instantiated with Conv2D() functions employing 3 × 3 kernels across 64, 128, and 512 filters. The convolutional layers, configured with 3 × 3 kernel sizes and a linear activation function, were systematically organized, with the Conv2D() function applied twice per dimension. Interposed with these are max-pooling layers, implemented via the MaxPooling2D() function, each with a 2 × 2 scope, to efficiently downscale feature dimensions.

The model harnesses the ReLU activation function, renowned for its efficacy in neural networks, particularly within CNN architectures. Following the convolutional sequences, the flatten() function transforms the network’s multidimensional output into a singular vector, seamlessly integrated with a densely connected layer. This terminal layer, equipped with a softmax activation function, is pivotal for resolving the classification dilemma inherent in this research. The inclusion of 256 neurons via the Dense() function, alongside a fully connected layer with a linear activation response, rounds off the model’s architecture. The Dense() layer is used to determine the image into one of two classes.

### 2.6. Computational Resources

The research was executed leveraging the Python 3.10 programming language, utilizing Jupyter Notebook and Google Colab Pro for the development environment on a personal computing device equipped with a Lenovo Yoga 11th Generation Intel(R) i7 processor. The deep learning experiments were facilitated using the Keras library [15], version 2.9.1, operating atop the TensorFlow [16] framework.

## 3. Results

**Figure 5:**
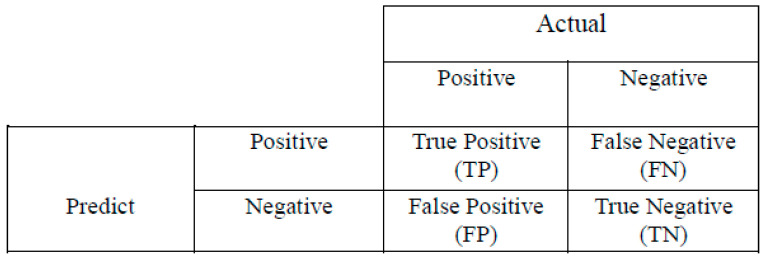
Confusion matrix.

To determine the efficacy of the trained models, we employed a suite of quantitative performance metrics, including the confusion matrix [17,18] (Figure 5), accuracy, precision, sensitivity, the F1 score, and loss, to evaluate their capabilities across assigned tasks [19,20]. Moreover, the time span of the training sessions was scrutinized. Definitions for the metrics used in our assessment are provided for a comprehensive understanding.

These metrics are predicated on the following definitions:

(1) $$Accuracy=\frac{\left(TP+TN\right)}{\left(TP+TN+FP+FN\right)}\times 100$$

(2) $$Precision=\frac{TP}{\left(TP+FP\right)}\times 100$$

(3) $$Recall=\frac{TP}{\left(TP+FN\right)}\times 100$$

(4) $$F1=\frac{TP}{\left(TP+TN+FP+FN\right)}\times 100$$

The definition of parameters used in (1)–(4) are given as follows:

- TP (true positive): The accurate identification of a true condition.
- FP (false positive): The erroneous identification of a condition as true.
- TN (true negative): The accurate identification of a false condition.
- FN (false negative): The erroneous identification of a condition as false.

The confusion matrix visualizes the correct and incorrect classifications of the model and helps us understand which class predictions are better or worse. Accuracy indicates the overall correct prediction rate of the model, whereas precision and sensitivity measure the quality of positive class predictions and the model’s ability to find true positives. The F1 score provides a combined assessment of these two measures, balancing precision and sensitivity. Loss indicates how effective the learning process is by reflecting the error rate in the model’s predictions. These multifaceted metrics allow us to analyze the performance of models in a more comprehensive way.

Given the inherent strengths and weaknesses of each model, selecting the optimal one for specific tasks or datasets becomes imperative. The commendable precision, recall, F1 score, and accuracy exhibited by most models signify their superior classification prowess, with EfficientNetB0 and ResNet50 demonstrating particularly outstanding overall performance.

Analysis of the data presented in Table 3 reveals EfficientNetB0 as the frontrunner in precision (92%), closely followed by ResNet50 and VGG16. This trend persists across recall rates and F1 scores, positioning EfficientNetB0 at the apex of model accuracy, a testament to its superior predictive capability.

**Figure 6:**
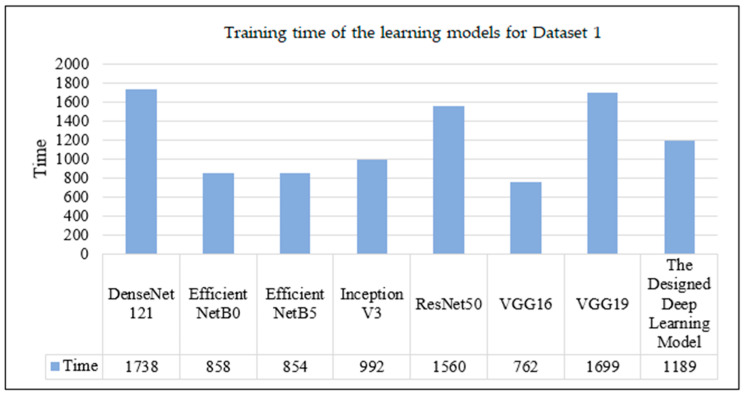
Training time of the learning models for Dataset 1.

The training duration varied significantly between models. As shown in Figure 6, VGG16 emerged as the most time-efficient model, requiring only 12 min 42 s (762 s) for training, whereas DenseNet121 recorded the longest training time of 28 min 58 s (1738 s).

An evaluation of the test results from Dataset 2 shows that VGG16 is a high-performance competitor, with a 92% success rate despite the long training time, as can be seen from the data in Table 4. On the other hand, ResNet50 stands out with an impressive 96% success rate, showcasing high precision, recall, F1 score, and accuracy, with its training time being relatively moderate in comparison. The developed deep learning model demonstrates moderate efficacy, with a training duration marginally exceeding that of its counterparts.

**Figure 7:**
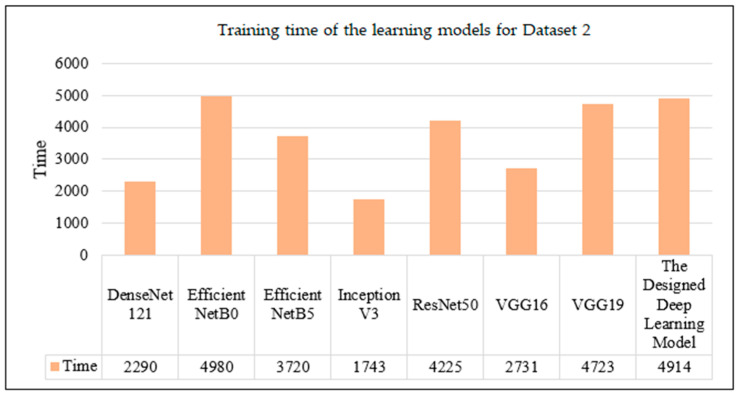
Training time of the learning models for Dataset 2.

Figure 7 illustrates that models like EfficientNetB0 and ResNet50 maintain superior performance even with prolonged training times. The custom model matches the performance of its peers while sustaining an average training duration.

The CNN, upon training and testing, attained 84% accuracy, 86% precision, 86% recall, and an 86% F1 score in Dataset 1. For Dataset 2, the figures were 86% for accuracy, 85% for precision, 84% for recall, and 84% for the F1 score. The training validation loss and accuracy for Datasets 1 and 2 are depicted through graphs, with the epoch value set at 10. Accuracy stood at 84% for Dataset 1 and 86% for Dataset 2, underscoring the network’s efficiency despite its relatively brief training span.

## 4. Discussion

Histopathology plays an important role in the diagnosis of diseases such as cancer. Deep learning algorithms are used as a powerful tool to identify pathological features in histopathological images and diagnose diseases. These algorithms are trained on large amounts of data in order to detect and analyze pre-defined features. The use of deep learning algorithms can speed up the diagnostic process for pathologists and help them achieve accurate results. In addition, this technology can reduce human error in image analysis and facilitate the early diagnosis of disease.

For the pathological diagnosis of pancreatic ductal adenocarcinoma, a 4:1 or more nuclear size change among ductal cells, incomplete ductal structures, and the irregular distribution of ducts as major criteria and single cell infiltration, irregular stroma, mitotic figures in epithelial cells, necrotic glandular debris, cribriform structures, large irregular eosinophilic nucleoli, and perineural invasion as minor criteria are helpful in reaching a diagnosis of malignancy [9]. In addition, the disruption of the normal lobular structure of the parenchyma and the presence of ductal structures in close proximity to large muscular vessels within the interlobular connective tissue, where they should not normally be found, are other helpful findings [21]. However, in the presence of significant atrophy, inflammation, and the irregular ductal structures observed in chronic pancreatitis, microscopic evaluation can be quite difficult. Especially in pancreatic resection materials showing findings of chronic pancreatitis, in order to exclude PDAC, a large number of samples must be obtained and a large number of slides must be evaluated in detail in terms of these morphological features, which is quite time-consuming and challenging. At this point, deep learning can assist the pathologist by detecting areas with suspicious findings, which can provide a diagnostic advantage in terms of reducing time loss for patients.

Kronberg et al. reported the detection of pancreatic cancer in hematoxylin and eosin-stained slides using deep transfer learning convolutional neural networks. The ResNet18 network achieved a five-class accuracy of approximately 94% on test data images. The network was validated on independent tissue sections consisting of healthy pancreatic tissue, pancreatic ductal adenocarcinoma (PDAC), and lymph node metastases from pancreatic cancer. Patient data were divided into 80% for training, 10% as normal, and 10% as test datasets. The raw dataset included 1690 images for the test data, 14,450 for training data, and 1702 for continuous data for a healthy pancreas; 874, 8275, and 805 slices for healthy lymph node (HLN) tissue; and 2454, 20,694, and 2502 slices for PDAC. The model achieved a weighted accuracy of 90%, a weighted Jaccard score of 81%, and a weighted F1 score of 90% across all classes. Individual F1 scores were 86% for a healthy pancreas and 92% for PDAC. The Jaccard scores were 82% for HLN and 85% for PDAC [2].

Zhang et al. conducted a retrospective multicenter diagnostic study using 5345 cytopathology slide images from 194 patients. They developed a deep convolutional neural network (DCNN) to segment cell clusters and identify cancer cells in cytopathology images. The DCNN achieved F1 scores of 0.929 in internal tests and 0.899–0.938 in external tests for separating stained cells from the background. For cancer identification, it showed an AUC of 0.958 internally and 0.948–0.976 externally. The DCNN’s performance surpassed that of validated endoscopists and cytopathologists in sensitivity analysis and test datasets, demonstrating its robustness and generalizability [22].

The study by Deepthi and colleagues demonstrated high accuracy for their models: VGG16 achieved 96%, ResNet 98%, and DenseNet 99%. Precision values were 95% for VGG16, 96% for ResNet, and 95% for DenseNet. Recall values were 98% for VGG16, 95% for ResNet, and 97% for DenseNet, whereas the F1 scores were 98%, 97%, and 98%, respectively. The findings indicate that VGG16, ResNet, and DenseNet are effective tools for pancreatic tumor detection, each with distinct advantages. By improving the early detection of pancreatic cancer, these models can enhance patient outcomes and inform future developments in medical image analysis [23].

Deep learning algorithms can therefore be used to diagnose histopathological images using a CNN model [24]. In this study, datasets were created using images of patients diagnosed with PDAC and CP from the Department of Pathology, Dokuz Eylul University Faculty of Medicine. The datasets were tested with DenseNet121, InceptionV3, EfficientNetB0, EfficientNetB5, ResNet50, VGG16, VGG19, and the designed network and analyzed to classify the models when extracting features from images.

**Figure 8:**
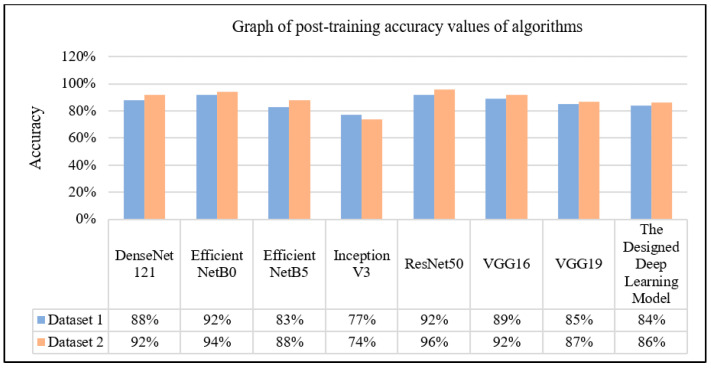
A comparison of the accuracy value of deep learning algorithms.

The models used in the study were evaluated in terms of precision, recall, the F1 score, and accuracy. In the graphs resulting from the training, it is clear that the EfficientNetB0 model performs better than the other models for Dataset 1. It achieved 92% for precision, recall, the F1 score, and accuracy. However, it is also clear that some models (e.g., InceptionV3) perform worse than others. From the analysis of training times, it can be seen that each model has a different training time. For example, the VGG16 model has a lower performance than the other models, although it has a shorter training time. The graphs resulting from the training of Dataset 1 compare the performance of different deep learning models and provide an idea of which model is preferable. In this context, the EfficientNetB0 model can be preferred as a better deep learning model than the others. In the graphs resulting from the training on Dataset 2, it is clearly seen that the ResNet50 and EfficientNetB0 models perform better than the other models. Both models achieved 94% and above for precision, recall, the F1 score, and accuracy. There are some differences between the other models. For instance, the Inceptionv3 model showed lower performance than the others, especially for precision, recall, and the F1-score, dropping to 75%. In the analysis of the training times, it can be seen that each of the models takes a different amount of time to train. For example, the EfficientNetB0 model was trained in only 1 h and 23 min but achieved high performance. The other models required longer training times. As a result, ResNet50 and EfficientNetB0 can be preferred as deep learning models for Dataset 2 (Figure 8). 

The deep learning algorithms used in the study are effective in identifying salient features to identify diseases by analyzing the histopathological dataset. This can be used to further improve the early detection of diseases such as cancer and determine appropriate treatment. However, there are some challenges associated with the use of deep learning classification algorithms.

In particular, the size and quality of datasets can affect the accuracy and generalizability of algorithms. For this reason, a dataset was created, and high-quality data was selected. To conclude, classifying histopathological images using deep learning has significant potential in the medical field. This technology can provide a faster, more accurate, and reliable approach to the diagnosis and treatment of diseases. To achieve accurate results, advanced data analysis and the continuous improvement of algorithms are required.

Furthermore, the training validation loss and accuracy plots show a steady improvement, providing information about the learning dynamics of the network. The high precision, recall, and F1 values obtained on both datasets underline the ability of the CNN to classify the data samples in an effective way. Overall, these results confirm the robustness and reliability of the CNN in processing different datasets and show its potential for real applications in different domains. 

## 5. Conclusions

In conclusion, our study shows that the classification performance for disease identification in PDAC tissue sections can be improved by preprocessing the dataset with different convolutional networks and by varying the hyperparameters of the designed network. Further studies on the application of this approach to metastases from different primary tumors will be necessary for validation.

## Figures and Tables

**Table 1:** The dataset created by the image cropping process and used in the study.

Dataset Information	Image Size	Number of Malignant Cell Images	Number of Beningn Cell Images
Dataset with cropping	256 × 256	6825	5670
	512 × 512	1365	1134
Dataset in the study	256 × 256	6000	6000
	512 × 512	2000	2000

**Table 2:** Distribution of Dataset 1 and Dataset 2 in the study.

Dataset	Train	Test	Validation
Dataset 1	9600	1440	960
Dataset 2	3200	480	320

**Table 3:** Testing results for Dataset 1.

Model	Precision	Accuracy	Recall	F1 Score	Loss	Training Time
DenseNet121	88%	88%	88%	88%	0.41	28 min 58 s
EfficientNetB0	92%	92%	92%	92%	0.40	14 min 18 s
EfficientNetB5	83%	83%	82%	82%	0.52	14 min 14 s
Inceptionv3	77%	77%	77%	77%	0.57	16 min 32 s
ResNet50	92%	92%	92%	92%	0.37	26 min
VGG16	89%	89%	88%	88%	0.41	12 min 42 s
VGG19	85%	85%	85%	85%	0.48	28 min 19 s
Designed Model	85%	84%	84%	84%	0.31	19 min 49 s

**Table 4:** Testing results for Dataset 2.

Model	Precision	Accuracy	Recall	F1 Score	Loss	Training Time
DenseNet121	92%	92%	92%	92%	0.43	38 min 10 s
EfficientNetB0	94%	94%	94%	94%	0.46	1 h 23 s
EfficientNetB5	88%	87%	87%	88%	0.67	1 h 2 s
Inceptionv3	75%	74%	74%	74%	0.90	29 min 3 s
ResNet50	96%	96%	96%	96%	0.40	1 h 10 min 25 s
VGG16	92%	92%	92%	92%	0.51	45 min 31 s
VGG19	87%	87%	87%	87%	0.56	1 h 18 min 43 s
Designed Model	86%	86%	86%	86%	0.32	1 h 21 min 54 s

## Data Availability

The data are available upon reasonable request from the authors.

## Abbreviations

CNN	Convolutional Neural Network
PDAC	Pancreatic Ductal Adenocarcinoma
CP	Chronic Pancreatitis
DNN	Deep Neural Network
RNN	Recurrent Neural Network
VGG	Visual Geometry Group
DENSENET	Densely Connected Convolutional Network
RESNET	Residual Network
ReLU	Rectified Linear Unit
TN	True Negative
TP	True Positive
FN	False Negative
FP	False Positive
HP	Healthy Pancreas
HLN	Healthy Lymph Node
DCNN	Deep Convolutional Neural Network
YCNN	YOLO Model-Based CNN
YOLO	You Only Look Once
